# Expression and Function of miR-155 in Diseases of the Gastrointestinal Tract

**DOI:** 10.3390/ijms17050709

**Published:** 2016-05-11

**Authors:** Jianhua Wan, Liang Xia, Wenting Xu, Nonghua Lu

**Affiliations:** Department of Gastroenterology, the First Affiliated Hospital of Nanchang University, Nanchang 330006, China; 18942337437@163.com (J.W.); liangx96180@126.com (L.X.); xuwentncdx@foxmail.com (W.X.)

**Keywords:** miR-155, *H. Pylori*, inflammatory bowel disease, colorectal cancer

## Abstract

MicroRNAs (miRNAs) are a type of small noncoding RNA that can regulate the expression of target genes under physiological and pathophysiological conditions. miR-155 is a multifunctional miRNA with inflammation-related and oncogenic roles. In particular, the dysregulation of miR-155 has been strongly implicated in *Helicobacter pylori*-related gastric disease, inflammatory bowel disease, and colorectal cancer in addition to being involved in molecular changes of important targets and signaling pathways. This review focuses on the expression and function of miR-155 during inflammation and carcinogenesis and its potential use as an effective therapeutic target for certain gastrointestinal diseases.

## 1. Introduction

MicroRNAs (miRNAs) are an abundant class of small noncoding RNAs that mostly down-regulate protein expression by either inducing mRNA degradation or suppressing protein synthesis. In 1993, the unique function of miRNAs was revealed for the first time in *Caenorhabditis elegans* by Lee *et al.* [1], who demonstrated regulation of lin-14 translation via an antisense RNA–RNA interaction. The precise structure of miRNAs determines their function. The biogenesis of miRNAs is a complex multi-step process involving many posttranscriptional modifications in the nucleus and cytoplasm. First, RNA polymerase II transcribes *miR* genes, generating primary miRNA (pri-miRNA). Next, pri-miRNA cleaves to the miRNA precursor (pre-miRNA) via the RNase III enzyme Drosha and the double-stranded-RNA-binding protein DGCR8 [2]. The cytoplasmic endonuclease Dicer cleaves the pre-miRNA stem-loop forming a 21–24 bp duplex miRNA. Next, one strand is loaded into the RNA-induced silencing complex (RISC) containing Argonaute (AGO) proteins [3,4,5]. The mature miRNA in the RISC then binds the 3′Untranslated Regions (3′UTR) of the target mRNAs and blocks their translation. Downstream target mRNAs of miRNAs play important roles in biological processes such as proliferation, differentiation, cellular migration, and apoptosis. Numerous reports indicate that miRNAs are associated with the posttranscriptional regulation of many proteins that are involved in numerous physiological and pathological processes [6,7], such as hematopoietic lineage differentiation, immunity, inflammation, cancer, and cardiovascular diseases [8].

MiR-155, an evolutionarily well-conserved miRNA that is mainly expressed in the thymus and spleen, is minimally detected in other tissues under normal physiological conditions. Transcription of miR-155 may be regulated by the activator protein-1 (AP-1) complex [9] and the nuclear factor-κB (NF-κB) transcription complex [10]. Target genes that are regulated by miR-155 include approximately 140 genes that encode for immunomodulatory proteins, tumor-suppressor proteins, and inflammatory-related proteins. Hence, ectopic miR-155 is often associated with specific disorders, including cardiovascular diseases, inflammation, and cancer [8]. Unlike the other miRNAs, miR-155 has been widely studied in the immune system under conditions of normal and abnormal immune responses and hematologic malignancies. In the gastrointestinal tract, abnormal miR-155 expression can be detected during *Helicobacter pylori* infection, and increased expression is observed in patients with inflammatory bowel disease (IBD) and colorectal cancer (CRC) [11,12,13]. However, miR-155-deficient mice not protected by immunization to *S. typhimurium* failed to produce significant levels of these cytokines [14]. Similarly, miR-155 is also essential for T-cell-mediated control of *H. pylori* infection [15]. Therefore, miR-155 may have unique functions in the immune-mediated process of certain gastrointestinal diseases.

## 2. Inflammation and Cancer

The inflammatory response is a complex process involving the induction of several inflammation-related genes and the activation of the immune system, which acts to clear pathogens and avoid pathological consequences. It is well-known that miR-155, as an important regulator, performs multiple functions in the immune system and is also required for normal immune function [14]. miR-155 expression in both myeloid and lymphoid cells following their activation is greatly increased in response to corresponding stimulation [9,16]. According to numerous recent studies, PU.1, AID, c-Maf, Bach1, Sla, Cutl1, Csf1r, Jarid2, Cebp,Arntl, Hif1α, Picalm, TAB2, SOCS1, SHIP-1, Rab6a, and others may act as functional targets of miR-155 in myeloid and lymphoid cells [14,17,18,19,20,21,22,23]. miR-155 has been identified and characterized as a component of the primary macrophage response to different types of inflammatory mediators such as bacterial lipopolysaccharide (LPS), interferon-β (IFN-β), polyriboinosinic-polyribocytidylic acid (poly IC), and tumor necrosis factor-α (TNF-α) [9]. Moreover, up-regulation of miR-155 is often associated with increased cytokine release during the inflammatory process [24]. Hence, miR-155, as a promoter of inflammatory responses, plays an important role by regulating the activation of immunocytes and the release of inflammatory cytokines. However, miR-155 can also control the intensity of the inflammatory response to microbial stimuli by targeting the Toll-like receptor/interleukin-1 (TLR/IL-1) inflammatory pathway in human dendritic cells (DCs) [20].

Recently, numerous studies have demonstrated that miR-155 is associated with hematological malignancies [25]. Transgenic miR-155 mice have also been shown to induce B cell malignancies *in vivo* [26]. With the exception of hematological malignancies, miR-155 can be detected in high levels in several solid tumors, such as CRC [12,13], lung cancer [13], breast cancer [13,27], thyroid carcinoma [28], cervical cancer [29], and pancreatic cancer [30,31], and it also serves as an indicator of a poor prognosis for patients with these tumors [32,33]. Several reports show that high levels of miR-155 are related to cancer cell invasion and poor survival [34,35]. At least 15%–20% of all human cancers are associated with chronic inflammation and diseases such as IBD (CRC and colitis-associated cancer), chronic gastritis, and *H. pylori* (gastric cancer, GC) [36]. miR-155, as a common target of a broad range of inflammatory mediators, may offer new insight into the mechanism of carcinoma. Hence, it is necessary to discuss the function of miR-155, which may act as a potential link between inflammation and cancer in the gastrointestinal tract.

## 3. *H. pylori*-Related Gastric Disease

*H. pylori* is a gram-negative spiral bacterium that was first described by Marshall and Warren in 1983 [37]. *H. pylori* acts as a primary pathogen because its presence in the stomach is almost always associated with a strong mucosal and systemic immune response [38]. *H. pylori* infection is often implicated in chronic gastritis, duodenal ulcer, gastric metaplasia, gastric extranodal marginal zone (MALT) lymphoma, and GC [39]. During *H. pylori* infection, miR-155 exhibits high expression levels in various cell types, including human T-cells, primary macrophages, and various epithelial cell lines, as well as in the gastric mucosa of infected mice and humans [11,15,40]. Similarly, miR-155 is up-regulated in *H. pylori*-positive gastritis [15], gastroduodenal ulcers [41,42], and MALT lymphoma [43,44]. In conclusion, *H. pylori* infection may be relevant to the induction of miR-155 expression.

**Figure 1:**
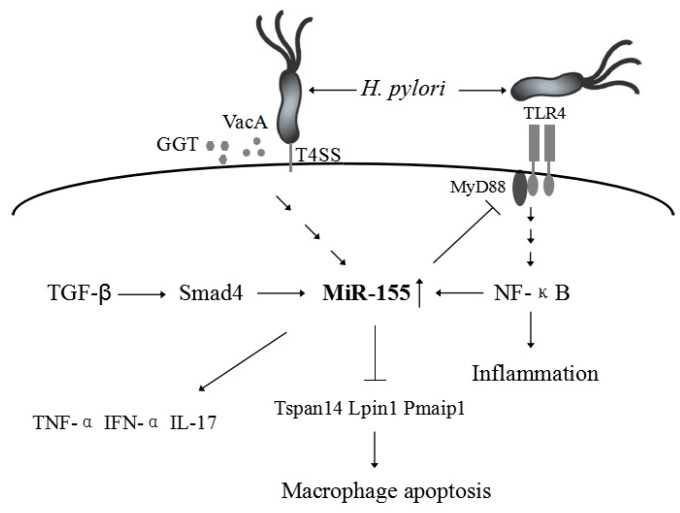
Overview of regulation of miR-155 by *H. pylori* infection.

During *H. pylori* infection, the gastric epithelial cells recognize the TLRs of the bacteria and then may activate a series of inflammation-related signaling pathways. *H. pylori* LPS, a cell wall component, is recognized mainly by TLR4 [45]. TLR4 can activate the myeloid differentiation primary-response gene 88 (MyD88), leading to translocation of NF-κB into the nucleus, subsequently activating the expression of genes related to the inflammatory process. Similarly, several TLR ligands have been shown to increase miR-155 expression via MyD88 [9]. Furthermore, up-regulation of miR-155 has been found to be dependent on the major *H. pylori* pathogenicity determinants, vacuolating toxin A (VacA), γ-glutamyl transpeptidase (GGT), and the type IV secretion system (T4SS) [40,46]. Related studies have indicated that *H. pylori* could induce miR-155 expression, which was dependent on the NF-κB and activator protein-1 (AP-1) pathway but was independent of CagA [9,11]. In addition, *H. pylori* can also induce miR-155 in T-cells in a cAMP-Foxp3-dependent manner [40]. However, Kong *et al.* [47] showed that TGF-β can induce miR-155 expression and promoter activity through Smad4. Thus, the regulation of miR-155 is a complex process that may involve co-regulation of multiple factors and signaling pathways (Figure 1).

Previous research has shown that *H. pylori* infections can be controlled by MHC class II-restricted Th1- and Th17-polarized Th cells in experimental murine models [48]. miR-155 has been shown to play a key role in T-cell-mediated control of *H. pylori* infections [15]. miR-155^−/−^ mice fail to control *H. pylori* infection as a result of impaired pathogen-specific Th1 and Th17 responses and exhibit significantly lower levels of gastric IFN-α and IL-17 production [15]. Similarly, overexpression of miR-155 by transfection of miR-155 mimics can significantly decrease the survival of intracellular *H. Pylori* [49]. Up-regulation of miR-155 can also lead to the expression of TNF-α [50]. miR-155 exhibits anti-apoptotic effects by targeting proapoptotic Tspan14, Lpin1, and Pmaip1, which could enhance the macrophage resistance to apoptosis induced by DNA damage during *H. pylori* infections [46]. Therefore, miR-155 is required for clearance of *H. pylori*. However, Xiao *et al.* [11] also noted that miR-155 may function as a negative regulator for the release of proinflammatory cytokines and signal transduction, which help to fine-tune the inflammatory response to *H. pylori* infection.

*H. pylori* infection is an important pathogenic factor in peptic ulcers; approximately 15%–20% of patients with a *H. pylori* infection develop peptic ulcer disease. However, all patients with a gastroduodenal ulcer are infected by *H. pylori* [51]. miR-155 is up-regulated during the development of *H. pylori*-infected gastroduodenal ulcers [41,42]. Cheng *et al.* [41] demonstrated that up-regulation of miR-155 and miR-146b decreased the over-expression of IL-6, impairing the immune response to *H. pylori* (cagA+) and contributing to the development of *H. pylori* (cagA+)-infected gastroduodenal ulcers. miR-155 may play a role in immune inhibition during gastroduodenal ulcer development, leading to chronic gastroduodenal inflammation and subsequent ulcer development.

Gastric MALT lymphomas are often associated with chronic inflammation caused by a *H. pylori* infection. Saito *et al.* [43] found that the expression levels of miR-155 in gastric MALT lymphoma lesions were significantly higher than those in the corresponding non-tumor gastric mucosae using miRNA microarray analysis and quantitative RT-PCR; this is associated with *H. pylori* eradication by targeting tumor protein p53-inducible nuclear protein 1(TP53INP), which induces cell cycle arrest and apoptosis. Therefore, the regulation and function of miR-155 in MALT lymphomas should continue to be evaluated.

GC is regarded as one of the most prevalent gastrointestinal cancers and has a low survival and high mortality rate. Development of gastric carcinoma involves multiple genetic lesions, such as oncogenes, tumor suppressor genes, and DNA mismatch repair genes [52,53]. Recently, many studies have demonstrated that miRNAs are closely correlated with GC development [54,55,56,57]. miRNAs have different expression levels in GC tissues that perform different functions. The expression levels of miR-155 in gastric carcinomas were significantly lower than those in the corresponding non-tumor gastric tissues [58]. Down-regulation of miR-155 accelerates cell growth and invasion by targeting c-myc in human gastric carcinoma cells [59]. c-myc serves as a pro-oncogene that is closely related to tumorigenesis and sustained tumor growth [60]. Thus, miR-155 may function as a gastric tumor suppressor during gastric carcinoma development. Similarly, Saito *et al.* [61] reported that up-regulation of miR-155 may inhibit GC cell metastasis by targeting Smad2. Thus, the regulation of miR-155 during gastric tumorigenesis is a complicated and poorly understood process.

Interestingly, during *H. pylori* infection, miR-155 exhibits increased expression in gastric mucosal tissues [11]; additionally, miR-155 is highly expressed during *H. pylori* positive gastritis [15], gastroduodenal ulcers [41,42], and MALT lymphoma [43,44]; however, miR-155 expression is reduced in GC cells and tissues [58,59]. miR-155 may play different roles in these diseases, and the process of gastric tumorigenesis is complex and may occur via silencing or loss of miR-155. Heterogeneity in biological functions could be another reason for these differences. Up-regulated miR-155 induced by *H. pylori* infections may contribute to the inflammatory response in *H. pylori*-related gastric disease.

## 4. Intestinal Diseases

The intestinal mucosal barrier plays an indispensable role in maintaining normalintestinal function and is composed of a layer of cross-linked, highly differentiated epithelial cells located at the apical junction complex (AJC). miR-155 over-expression inhibits RhoA protein expression, which down-regulates the expression of zonula occludens-1 (ZO-1) and *E*-cadherin, two major component proteins of the AJC [62]. Many inflammatory cytokines, such as TNF-α and interferons, also reduce the mucosal barrier function by destroying the AJC [63,64]. Similarly, miR-155 expression can be induced by TNF-α and interferons [9,50,65,66]. Thus, the regulation of miR-155 plays a key role in intestinal mucosal barrier function, which is closely associated with intestinal diseases.

IBD refers to chronic inflammatory disorders of the gut with unclear pathogenesis, including Crohn’s disease (CD) and ulcerative colitis (UC) [67]. IBD is characterized by underlying immunological deregulation, a susceptible genetic background, and intestinal microbes that lead to damage of the intestinal mucosa [68,69]. In IBD, mucosal inflammation may be caused by an intestinal barrier injury, which may result from increasing permeability and destruction of the tight junctions [70].

Deregulated miRNA expression is involved in a variety of disorders, such as IBD [71,72]. Several studies have shown that the expression of miR-155 is increased in IBDs including UC and CD [73,74,75]. When miRNA expression profiles were analyzed in patients with active UC, miR-155 was up-regulated in patients with active UC compared to healthy subjects, resulting in a direct down-regulation of the expression of Forkhead box O3 (FOXO3a), which may activate the NF-κB signaling pathway and up-regulate inflammatory cytokines such as IL-8 [76,77]. The pathogenic mechanism of UC, which may involve multiple immune process and immunological inflammation, is currently unclear. The inflammatory process of the intestinal mucosa involves many intestinal stromal cells such as mesenchymal cells, including intestinal fibroblasts and myofibroblasts (IMFs) [78]. In UC, miR-155 modulates the inflammatory phenotype of IMFs by targeting the suppressor of cytokine signaling 1 (SOCS1), which is a known feedback inhibitor of inflammation [79]. Thus, this is an effective approach that results in decreasing deregulated cytokine release from IMFs by suppressing miR-155. Previous studies have indicated that T-cell-mediated injury may be a pathogenic pathway for the intestinal damage associated with IBD [80]. Loss of miR-155 protects mice from experimental colitis, which involves multiple pathways, including decreased TNF-α, IL-6, IL-12, IL-17, and IFN-γ production, decreased Th1 responses and reduced activation of T-cells by DCs [81]. Inhibition of the target of miR-155 can diminish the release of inflammatory cytokines and reduce the severity of colitis. This finding shows that miR-155 inhibition may be a novel and effective therapeutic target for colitis and IBD.

CRC was the third most commonly-diagnosed cancer in males and the second most commonly diagnosed cancer in females in 2012, and its incidence is increasing in several Asian and Eastern European countries [82]. Although the development of CRC therapy is proceeding at a fast pace, the prognosis of advanced CRC is still very poor. Thus, clarifying the pathogenesis of CRC is highly desirable. A relationship between miRNA function and cancer pathogenesis has been demonstrated. In real-time PCR analysis, many miRNAs exhibit different expression levels in CRC and non-tumoral tissues [83,84]. Similarly, over-expression of miR-155 has been detected in CRC [12,13]. Qu *et al.* [12] noted that miR-155 could promote proliferation, invasion, and metastasis of CRC cells; its up-regulation was closely related to tumor location, tumor grade, Tumor Node Metastasis (TNM) staging, and distant metastasis in CRC. In general, the mismatch repair (MMR) system is widely present in many organisms, acts as the repair mechanism after cell replication, and maintains the fidelity of DNA replication. miR-155 causes a decrease in MMR protein stability and down-regulates core MMR proteins in the MMR system in CRC, thus inducing microsatellite instability (MSI) [85]. MSI is divided into three types: high levels of MSI (MSI-H), low levels of MSI (MSI-L) and microsatellite stability (MSS). In CRC, increased relative expression of miR-155 is more significantly associated with MSI-H status than with MSI-L and MSS status [86]. Similarly, miR-155 overexpression is particularly associated with MSI in IBD and CRCs and extends to the distant non-neoplastic mucosa [87]. In summary, miR-155 over-expression may induce MSI by regulating the MMR system, thus promoting MSI-driven neoplastic transformation of the colonic mucosa. However, miR-155 over-expression may play a role in oncogenesis by combining with inflammatory stimuli in tumor cells. These changes may indicate the potential pathogenetic mechanism of CRC. Further research is needed to identify an intervention for this mechanism.

## 5. Discussion and Conclusions

miR-155 can regulate innate and adaptive immunity, which control antibody production and cytokine release. Although regulation of miR-155 has been widely studied in the innate immune process and immune-mediated diseases, it has also recently become the focus of study for various malignancies. In this review, we report the expression and function of miR-155 in gastrointestinal diseases such as *H. pylori*-related gastric disease, IBD, and CRC (Table 1). miR-155 may be a suitable choice for use as an interesting and innovative therapeutic target or a biomarker of disease type and severity for certain human cancers. However, a recent study found that increased miR-155 expression was correlated with an improved prognosis in human melanoma patients [88]. This condition may explain the dysregulation of miR-155, which may be a direct negative regulator of GC cell proliferation and survival in GC patients. However, the exact carcinogenic mechanism of miR-155 is not entirely clear; therefore, more studies that lead to an understanding of the role and pathogenic mechanisms of miR-155 are necessary to search for effective treatment modalities for these gastrointestinal diseases. miR-155 may be an attractive candidate for the development of new therapeutic interventions in the near future.

## Figures and Tables

**Table 1:** Role of miR-155 in diseases of the gastrointestinal tract.

Disease	Expression	Function	Target(s)
***H. pylori*-related gastric disease**			
*H. pylori* infection	Up-regulation	Decreases the survival of intracellular *H. Pylori* [49], induces the expression of TNF-α [50], enhances the macrophage resistance to apoptosis [46], or reduces the release of proinflammatory cytokines and signal transduction [11].	Tspan14, Lpin1, and Pmaip1 [46]
Peptic ulcers	Up-regulation	Decreases the over-expression of IL-6 and impairs the immune response to H. pylori (cagA+) [41].	
Gastric MALT lymphomas	Up-regulation	Induces cell cycle arrest and apoptosis [43].	TP53INP [43]
GC	Down-regulation	Accelerates cell growth and invasion in human gastric carcinoma cells [59], inhibit GC cell metastasis [61].	c-myc [59], Smad2 [61]
**Intestinal diseases**			
IBD	Up-regulation	Activates the NF-κB signaling pathway and up-regulate inflammatory cytokines such as IL-8 [76], modulates the inflammatory phenotype of IMFs [79], Loss of miR-155 protects mice from experimental colitis by decreasing TNF-(IL-6, IL-12, IL-17, and IFN-γ production [81].	FOXO3a [76], SOCS1 [79]
CRC	Up-regulation	Promotes proliferation, invasion, and metastasis of CRC cells, is closely related to tumor location, tumor grade, TNM staging, and distant metastasis in CRC [12], induces MSI by regulating the MMR system [85].	

## Abbreviations

AJC	apical junction complex
CRC	colorectal cancer
CD	Crohn’s disease
GC	gastric cancer
GGT	γ-glutamyl transpeptidase
IBD	inflammatory bowel disease
IMFs	intestinal fibroblasts and myofibroblasts
MMR	mismatch repair
MSI	microsatellite instability
MyD88	myeloid differentiation primary-response gene 88
poly IC	polyriboinosinic-polyribocytidylic acid
RISC	RNA-induced silencing complex
SOCS1	suppressor of cytokine signaling 1
T4SS	type IV secretion system
TLR/IL-1	Toll-like receptor/interleukin-1
TP53INP	tumor protein p53-inducible nuclear protein 1
UC	ulcerative colitis
VacA	vacuolating toxin A

## References

[1] Lee R.C., Feinbaum R.L., Ambros V. (1993). The *C. elegans* heterochronic gene *lin-4* encodes small RNAs with antisense complementarity to *lin-14*. Cell.

[2] Denli A.M., Tops B.B., Plasterk R.H., Ketting R.F., Hannon G.J. (2004). Processing of primary microRNAs by the Microprocessor complex. Nature.

[3] Chendrimada T.P., Gregory R.I., Kumaraswamy E., Norman J., Cooch N., Nishikura K., Shiekhattar R. (2005). TRBP recruits the Dicer complex to Ago2 for microRNA processing and gene silencing. Nature.

[4] Diederichs S., Haber D.A. (2007). Dual role for argonautes in microRNA processing and posttranscriptional regulation of microRNA expression. Cell.

[5] Rana T.M. (2007). Illuminating the silence: Understanding the structure and function of small RNAs. Nat. Rev. Mol. Cell Biol..

[6] Ambros V. (2004). The functions of animal microRNAs. Nature.

[7] Bartel D.P. (2004). MicroRNAs: Genomics, biogenesis, mechanism, and function. Cell.

[8] Faraoni I., Antonetti F.R., Cardone J., Bonmassar E. (2009). *miR-155* gene: A typical multifunctional microRNA. Biochim. Biophys. Acta.

[9] O′Connell R.M., Taganov K.D., Boldin M.P., Cheng G., Baltimore D. (2007). MicroRNA-155 is induced during the macrophage inflammatory response. Proc. Natl. Acad. Sci. USA.

[10] Thai T.H., Calado D.P., Casola S., Ansel K.M., Xiao C., Xue Y., Murphy A., Frendewey D., Valenzuela D., Kutok J.L. (2007). Regulation of the germinal center response by microRNA-155. Science.

[11] Xiao B., Liu Z., Li B.S., Tang B., Li W., Guo G., Shi Y., Wang F., Wu Y., Tong W.D. (2009). Induction of microRNA-155 during *Helicobacter pylori* infection and its negative regulatory role in the inflammatory response. J. Infect. Dis..

[12] Qu Y.L., Wang H.F., Sun Z.Q., Tang Y., Han X.N., Yu X.B., Liu K. (2015). Up-regulated miR-155–5p promotes cell proliferation, invasion and metastasis in colorectal carcinoma. Int. J. Clin. Exp. Pathol..

[13] Volinia S., Calin G.A., Liu C.G., Ambs S., Cimmino A., Petrocca F., Visone R., Iorio M., Roldo C., Ferracin M. (2006). A microRNA expression signature of human solid tumors defines cancer gene targets. Proc. Natl. Acad. Sci. USA.

[14] Rodriguez A., Vigorito E., Clare S., Warren M.V., Couttet P., Soond D.R., van Dongen S., Grocock R.J., Das P.P., Miska E.A. (2007). Requirement of *bic/microRNA-155* for normal immune function. Science.

[15] Oertli M., Engler D.B., Kohler E., Koch M., Meyer T.F., Muller A. (2011). MicroRNA-155 is essential for the T cell-mediated control of Helicobacter pyloriinfection and for the induction of chronic Gastritis and Colitis. J. Immunol..

[16] Haasch D., Chen Y.W., Reilly R.M., Chiou X.G., Koterski S., Smith M.L., Kroeger P., McWeeny K., Halbert D.N., Mollison K.W. (2002). T cell activation induces a noncoding RNA transcript sensitive to inhibition by immunosuppressant drugs and encoded by the proto-oncogene, *BIC*. Cell. Immunol..

[17] O′Connell R.M., Chaudhuri A.A., Rao D.S., Baltimore D. (2009). Inositol phosphatase SHIP1 is a primary target of miR-155. Proc. Natl. Acad. Sci. USA.

[18] Vigorito E., Perks K.L., Abreu-Goodger C., Bunting S., Xiang Z., Kohlhaas S., Das P.P., Miska E.A., Rodriguez A., Bradley A. (2007). microRNA-155 regulates the generation of immunoglobulin class-switched plasma cells. Immunity.

[19] Lu L.F., Thai T.H., Calado D.P., Chaudhry A., Kubo M., Tanaka K., Loeb G.B., Lee H., Yoshimura A., Rajewsky K. (2009). Foxp3-dependent microRNA155 confers competitive fitness to regulatory T cells bytargeting SOCS1 protein. Immunity.

[20] Ceppi M., Pereira P.M., Dunand-Sauthier I., Barras E., Reith W., Santos M.A., Pierre P. (2009). MicroRNA-155 modulates the interleukin-1 signaling pathway in activated human monocyte-derived dendritic cells. Proc. Natl. Acad. Sci. USA.

[21] Dorsett Y., McBride K.M., Jankovic M., Gazumyan A., Thai T.H., Robbiani D.F., di Virgilio M., Reina S.B., Heidkamp G., Schwickert T.A. (2008). MicroRNA-155 suppresses activation-induced cytidine deaminase-mediated Myc-Igh translocation. Immunity.

[22] Teng G., Hakimpour P., Landgraf P., Rice A., Tuschl T., Casellas R., Papavasiliou F.N. (2008). MicroRNA-155 is a negative regulator of activation-induced cytidine deaminase. Immunity.

[23] Yang Y., Yang L. (2015). Identification of Rab6a as a new target of microRNA-155 involved in regulating lipopolysaccharide-induced TNF secretion. Inflammation.

[24] Cremer T.J., Ravneberg D.H., Clay C.D., Piper-Hunter M.G., Marsh C.B., Elton T.S., Gunn J.S., Amer A., Kanneganti T.D., Schlesinger L.S. (2009). MiR-155 induction by *F. novicida* but not the virulent *F. tularensis* results in SHIP down-regulation and enhanced pro-inflammatory cytokine response. PLoS ONE.

[25] Ranganath P. (2015). MicroRNA-155 and its role in malignant hematopoiesis. Biomark. Insights.

[26] Costinean S., Zanesi N., Pekarsky Y., Tili E., Volinia S., Heerema N., Croce C.M. (2006). Pre-B cell proliferation and lymphoblastic leukemia/high-grade lymphoma in E(mu)-miR155 transgenic mice. Proc. Natl. Acad. Sci. USA.

[27] Iorio M.V., Ferracin M., Liu C.G., Veronese A., Spizzo R., Sabbioni S., Magri E., Pedriali M., Fabbri M., Campiglio M. (2005). MicroRNA gene expression deregulation in human breast cancer. Cancer Res..

[28] Nikiforova M.N., Tseng G.C., Steward D., Diorio D., Nikiforov Y.E. (2008). MicroRNA expression profiling of thyroid tumors: Biological significance and diagnostic utility. J. Clin. Endocrinol. Metab..

[29] Wang X., Tang S., Le S.-Y., Lu R., Rader J.S., Meyers C., Zheng Z.M. (2008). Aberrant expression of oncogenic and tumor-suppressive microRNAs in cervical cancer is required for cancer cell growth. PLoS ONE.

[30] Lee E.J., Gusev Y., Jiang J., Nuovo G.J., Lerner M.R., Frankel W.L., Morgan D.L., Postier R.G., Brackett D.J., Schmittgen T.D. (2007). Expression profiling identifies microRNA signature in pancreatic cancer. Int. J. Cancer.

[31] Szafranska A.E., Davison T.S., John J., Cannon T., Sipos B., Maghnouj A., Labourier E., Hahn S.A. (2007). MicroRNA expression alterations are linked to tumorigenesis and non-neoplastic processes in pancreatic ductal adenocarcinoma. Oncogene.

[32] Jay C., Nemunaitis J., Chen P., Fulgham P., Tong A.W. (2007). miRNA profiling for diagnosis and prognosis of human cancer. DNA Cell Biol..

[33] Yanaihara N., Caplen N., Bowman E., Seike M., Kumamoto K., Yi M., Stephens R.M., Okamoto A., Yokota J., Tanaka T. (2006). Unique microRNA molecular profiles in lung cancer diagnosis and prognosis. Cancer Cell.

[34] Chang S., Wang R.H., Akagi K., Kim K.A., Martin B.K., Cavallone L., Haines D.C., Basik M., Mai P., Poggi E. (2011). Tumor suppressor BRCA1 epigenetically controls oncogenic microRNA-155. Nat. Med..

[35] Han Z.B., Chen H.Y., Fan J.W., Wu J.Y., Tang H.M., Peng Z.H. (2012). Up-regulation of microRNA-155 promotes cancer cell invasion and predicts poor survival of hepatocellular carcinoma following liver transplantation. J. Cancer Res. Clin. Oncol..

[36] Rath T., Billmeier U., Waldner M.J., Atreya R., Neurath M.F. (2015). From physiology to disease and targeted therapy: Interleukin-6 in inflammation and inflammation-associated carcinogenesis. Arch. Toxicol..

[37] Marshall B.J., Warren J.R. (1984). Unidentified curved bacilli in the stomach of patients with gastritis and pepticulceration. Lancet.

[38] Figura N. (1997). Identifiable *Helicobacter pylori* strains or factors important in the developmentof duodenal ulcer disease. Helicobacter.

[39] Wroblewski L.E., Peek R.J. (2013). *Helicobacter pylori* in gastric carcinogenesis: Mechanisms. Gastroenterol. Clin. N. Am..

[40] Fassi F.L., Koch M., Belogolova E., Khalil H., Bolz C., Kalali B., Mollenkopf H.J., Beigier-Bompadre M., Karlas A., Schneider T. (2010). *Helicobacter pylori* induces miR-155 in T cells in a cAMP-Foxp3-dependent manner. PLoS ONE.

[41] Cheng S.F., Li L., Wang L.M. (2015). miR-155 and miR-146b negatively regulates IL6 in *Helicobacter pylori* (cagA+) infected gastroduodenal ulcer. Eur. Rev. Med. Pharmacol. Sci..

[42] Lario S., Ramirez-Lazaro M.J., Aransay A.M., Lozano J.J., Montserrat A., Casalots A., Junquera F., Alvarez J., Segura F., Campo R. (2012). microRNA profiling in duodenal ulcer disease caused by *Helicobacter pylori* infection in a Western population. Clin. Microbiol. Infect..

[43] Saito Y., Suzuki H., Tsugawa H., Imaeda H., Matsuzaki J., Hirata K., Hosoe N., Nakamura M., Mukai M., Saito H. (2012). Overexpression of miR-142-5p and miR-155 in gastric mucosa-associated lymphoid tissue (MALT) lymphoma resistant to *Helicobacter pylori* eradication. PLoS ONE.

[44] Thorns C., Kuba J., Bernard V., Senft A., Szymczak S., Feller A.C., Bernd H. (2012). Deregulation of a distinct set of microRNAs is associated with transformation of gastritis into MALT lymphoma. Virchows Arch..

[45] Matsuura M. (2013). Structural modifications of bacterial lipopolysaccharide that facilitate gram-negative bacteria evasion of host innate immunity. Front. Immunol..

[46] Koch M., Mollenkopf H.J., Klemm U., Meyer T.F. (2012). Induction of microRNA-155 is TLR- and type IV secretion system-dependent in macrophages and inhibits DNA-damage induced apoptosis. Proc. Natl. Acad. Sci. USA.

[47] Kong W., Yang H., He L., Zhao J.J., Coppola D., Dalton W.S., Cheng J.Q. (2008). MicroRNA-155 is regulated by the transforming growth factor β/Smad pathway and contributes to epithelial cell plasticity by targeting RhoA. Mol. Cell. Biol..

[48] Hitzler I., Oertli M., Becher B., Agger E.M., Muller A. (2011). Dendritic cells prevent rather than promote immunity conferred by a helicobactervaccine using a mycobacterial adjuvant. Gastroenterology.

[49] Wu K., Zhu C., Yao Y., Wang X., Song J., Zhai J. (2016). MicroRNA-155-enhanced autophagy in human gastric epithelial cell in response to *Helicobacter pylori*. Saudi J. Gastroenterol..

[50] Tili E., Michaille J.J., Cimino A., Costinean S., Dumitru C.D., Adair B., Fabbri M., Alder H., Liu C.G., Calin G.A. (2007). Modulation of miR-155 and miR-125b levels following lipopolysaccharide/TNF-α stimulation and their possible roles in regulating the response to endotoxin shock. J. Immunol..

[51] Ateshkadi A., Lam N.P., Johnson C.A. (1993). *Helicobacter pylori* and peptic ulcer disease. Clin. Pharm..

[52] Yasui W., Yokozaki H., Fujimoto J., Naka K., Kuniyasu H., Tahara E. (2000). Genetic and epigenetic alterations in multistep carcinogenesis of the stomach. J. Gastroenterol..

[53] Tahara E. (1995). Molecular biology of gastric cancer. World J. Surg..

[54] Liu Y., Xing R., Zhang X., Dong W., Zhang J., Yan Z., Li W., Cui J., Lu Y. (2013). miR-375 targets the *p53* gene to regulate cellular response to ionizing radiationand etoposide in gastric cancer cells. DNA Repair..

[55] Yang T.S., Yang X.H., Wang X.D., Wang Y.L., Zhou B., Song Z.S. (2013). MiR-214 regulate gastric cancer cell proliferation, migration and invasion by targeting PTEN. Cancer Cell Int..

[56] Sacconi A., Biagioni F., Canu V., Mori F., Di Benedetto A., Lorenzon L., Ercolani C., di Agostino S., Cambria A.M., Germoni S. (2012). miR-204 targets Bcl-2 expression and enhances responsiveness of gastric cancer. Cell. Death Dis..

[57] Wang J., Song Y., Wang Z. (2015). Non-coding RNAs in gastric cancer. Gene.

[58] Li H., Xie S., Liu M., Chen Z., Liu X., Wang L., Li D., Zhou Y. (2014). The clinical significance of downregulation of miR-124-3p, miR-146a-5p, miR-155-5p and miR-335-5p in gastric cancer tumorigenesis. Int. J. Oncol..

[59] Sun S., Sun P., Wang C., Sun T. (2014). Downregulation of microRNA-155 accelerates cell growth and invasion by targetingc-myc in human gastric carcinoma cells. Oncol. Rep..

[60] Lin C.Y., Loven J., Rahl P.B., Paranal R.M., Burge C.B., Bradner J.E., Lee T.I., Young R.A. (2012). Transcriptional amplification in tumor cells with elevated c-Myc. Cell.

[61] Li C.L., Nie H., Wang M., Su L.P., Li J.F., Yu Y.Y., Yan M., Qu Q.L., Zhu Z.G., Liu B.Y. (2012). microRNA-155 is downregulated in gastric cancer cells and involved in cell metastasis. Oncol. Rep..

[62] Tian R. (2013). Overexpressed miRNA-155 dysregulates intestinal epithelial apical junctional complex in severe acute pancreatitis. World J. Gastroenterol..

[63] Sasaki M., Sitaraman S.V., Babbin B.A., Gerner-Smidt P., Ribot E.M., Garrett N., Alpern J.A., Akyildiz A., Theiss A.L., Nusrat A. (2007). Invasive *Escherichia coli* are a feature of Crohn’s disease. Lab. Investig..

[64] Fries W., Muja C., Crisafulli C., Cuzzocrea S., Mazzon E. (2008). Dynamics of enterocyte tight junctions: Effect of experimental colitis and two different anti-TNF strategies. Am. J. Physiol. Gastrointest. Liver Physiol..

[65] Imaizumi T., Tanaka H., Tajima A., Yokono Y., Matsumiya T., Yoshida H., Tsuruga K., Aizawa-Yashiro T., Hayakari R., Inoue I. (2010). IFN-γ and TNF-α synergistically induce microRNA-155 which regulates TAB2/IP-10 expression in human mesangial cells. Am. J. Nephrol..

[66] Moschos S.A., Williams A.E., Perry M.M., Birrell M.A., Belvisi M.G., Lindsay M.A. (2007). Expression profiling *in vivo* demonstrates rapid changes in lung microRNA levels following lipopolysaccharide-induced inflammation but not in the anti-inflammatory action of glucocorticoids. BMC Genom..

[67] Kaser A., Zeissig S., Blumberg R.S. (2010). Inflammatory bowel disease. Annu. Rev. Immunol..

[68] Fiocchi C. (1998). Inflammatory bowel disease: Etiology and pathogenesis. Gastroenterology.

[69] Abraham C., Cho J.H. (2009). Inflammatory bowel disease. N. Engl. J. Med..

[70] Turner J.R. (2006). Molecular basis of epithelial barrier regulation. Am. J. Pathol..

[71] Coskun M., Bjerrum J.T., Seidelin J.B., Nielsen O.H. (2012). MicroRNAs in inflammatory bowel disease—Pathogenesis, diagnostics and therapeutics. World J. Gastroenterol..

[72] Kalla R., Ventham N.T., Kennedy N.A., Quintana J.F., Nimmo E.R., Buck A.H., Satsangi J. (2015). MicroRNAs: New players in IBD. Gut.

[73] Fasseu M., Treton X., Guichard C., Pedruzzi E., Cazals-Hatem D., Richard C., Aparicio T., Daniel F., Soule J.C., Moreau R. (2010). Identification of restricted subsets of mature microRNA abnormally expressed in inactive colonic mucosa of patients with inflammatory bowel disease. PLoS ONE.

[74] Takagi T., Naito Y., Mizushima K., Hirata I., Yagi N., Tomatsuri N., Ando T., Oyamada Y., Isozaki Y., Hongo H. (2010). Increased expression of microRNA in the inflamed colonic mucosa of patients withactive ulcerative colitis. J. Gastroenterol. Hepatol..

[75] Wu F., Zhang S., Dassopoulos T., Harris M.L., Bayless T.M., Meltzer S.J., Brant S.R., Kwon J.H. (2010). Identification of microRNAs associated with ileal and colonic Crohn’s disease. Inflamm. Bowel Dis..

[76] Min M., Peng L., Yang Y., Guo M., Wang W., Sun G. (2014). MicroRNA-155 is involved in the pathogenesis of ulcerative colitis by targeting FOXO3a. Inflamm. Bowel Dis..

[77] Lin L., Hron J.D., Peng S.L. (2004). Regulation of NF-κB, Th activation, and autoinflammation by the forkhead transcription factor FOXO3a. Immunity.

[78] Owens B.M., Simmons A. (2013). Intestinal stromal cells in mucosal immunity and homeostasis. Mucosal Immunol..

[79] Pathak S., Grillo A.R., Scarpa M., Brun P., D’Incà R., Nai L., Banerjee A., Cavallo D., Barzon L., Palù G. (2015). MiR-155 modulates the inflammatory phenotype of intestinal myofibroblasts by targeting SOCS1 in ulcerative colitis. Exp. Mol. Med..

[80] Elson C.O., Beagley K.W., Sharmanov A.T., Fujihashi K., Kiyono H., Tennyson G.S., Cong Y., Black C.A., Ridwan B.W., McGhee J.R. (1996). Hapten-induced model of murine inflammatory bowel disease: Mucosa immune responses and protection by tolerance. J. Immunol..

[81] Singh U.P., Murphy A.E., Enos R.T., Shamran H.A., Singh N.P., Guan H., Hegde V.L., Fan D., Price R.L., Taub D.D. (2014). miR-155 deficiency protects mice from experimental colitis by reducing T helper type 1/type 17 responses. Immunology.

[82] Torre L.A., Bray F., Siegel R.L., Ferlay J., Lortet-Tieulent J., Jemal A. (2015). Global cancer statistics, 2012. CA Cancer J. Clin..

[83] Bandres E., Cubedo E., Agirre X., Malumbres R., Zarate R., Ramirez N., Abajo A., Navarro A., Moreno I., Monzo M. (2006). Identification by Real-time PCR of 13 mature microRNAs differentially expressed in colorectal cancer and non-tumoral tissues. Mol. Cancer.

[84] Ng E.K., Chong W.W., Jin H., Lam E.K., Shin V.Y., Yu J., Poon T.C., Ng S.S., Sung J.J. (2009). Differential expression of microRNAs in plasma of patients with colorectal cancer: A potential marker for colorectal cancer screening. Gut.

[85] Valeri N., Gasparini P., Fabbri M., Braconi C., Veronese A., Lovat F., Adair B., Vannini I., Fanini F., Bottoni A. (2010). Modulation of mismatch repair and genomic stability by miR-155. Proc. Natl. Acad. Sci. USA.

[86] Earle J.S., Luthra R., Romans A., Abraham R., Ensor J., Yao H., Hamilton S.R. (2010). Association of microRNA expression with microsatellite instability status in colorectal adenocarcinoma. J. Mol. Diagn..

[87] Svrcek M., El-Murr N., Wanherdrick K., Dumont S., Beaugerie L., Cosnes J., Colombel J.F., Tiret E., Flejou J.F., Lesuffleur T. (2013). Overexpression of microRNAs-155 and 21 targeting mismatch repair proteins in inflammatory bowel diseases. Carcinogenesis.

[88] Segura M.F., Belitskaya-Levy I., Rose A.E., Zakrzewski J., Gaziel A., Hanniford D., Darvishian F., Berman R.S., Shapiro R.L., Pavlick A.C. (2010). Melanoma MicroRNA signature predicts post-recurrence survival. Clin. Cancer Res..

